# Anomalous Fluorescence Enhancement from Double Heterostructure 3D Colloidal Photonic Crystals–A Multifunctional Fluorescence-Based Sensor Platform

**DOI:** 10.1038/srep14439

**Published:** 2015-09-24

**Authors:** Ehsan Eftekhari, Xiang Li, Tak H. Kim, Zongsong Gan, Ivan S. Cole, Dongyuan Zhao, Dave Kielpinski, Min Gu, Qin Li

**Affiliations:** 1 Queensland Micro- and Nanotechnology Centre & School of Engineering, Griffith University, Nathan, QLD 4111, Australia; 2 CSIRO Materials Science and Engineering, Clayton, VIC 3168, Australia; 3 Department of Chemistry & Laboratory of Advanced Materials, Fudan University, Shanghai, 200433, P.R. China; 4 Centre for Quantum Dynamics, Griffith University, Nathan, QLD 4111, Australia; 5 Centre for Micro-Photonics and CUDOS, Faculty of Science, Engineering and Technology, Swinburne University of Technology, Hawthorn, VIC 3122, Australia

## Abstract

Augmenting fluorescence intensity is of vital importance to the development of chemical and biochemical sensing, imaging and miniature light sources. Here we report an unprecedented fluorescence enhancement with a novel architecture of multilayer three-dimensional colloidal photonic crystals self-assembled from polystyrene spheres. The new technique uses a double heterostructure, which comprises a top and a bottom layer with a periodicity overlapping the excitation wavelength (E) of the emitters, and a middle layer with a periodicity matching the fluorescence wavelength (F) and a thickness that supports constructive interference for the excitation wavelength. This E-F-E double heterostructure displays direction-dependent light trapping for both excitation and fluorescence, coupling the modes of photonic crystal with multiple-beam interference. The E-F-E double heterostructure renders an additional 5-fold enhancement to the extraordinary FL amplification of Rhodamine B in monolithic E CPhCs, and 4.3-fold acceleration of emission dynamics. Such a self-assembled double heterostructue CPhCs may find significant applications in illumination, laser, chemical/biochemical sensing, and solar energy harvesting. We further demonstrate the multi-functionality of the E-F-E double heterostructure CPhCs in Hg (II) sensing.

Signal intensity and signal-to-noise ratio are the defining features for molecular and nanoparticle luminescence, which is of vital importance to technological development in chemical/biochemical sensing^1^, disease diagnostics^2^, environmental monitoring^3^, light emitting devices^4^ and optoelectronics^5^. Recently there has been a growing interest in employing photonic crystals (PhCs) for manipulating luminescent emissions^6^,^7^,^8^.

PhCs are artificial periodic structures consisting of different dielectric materials where the index of refraction varies on length scales of the wavelength of light. The periodic dielectrics contrast induces a forbidden region for electromagnetic waves, namely the photonic bandgap, or stopband for partial bandgap, which provides powerful means to control light^9^,^10^. PhCs can be conveniently fabricated by bottom-up self-assembly with monodisperse submicron-particles, called colloidal PhCs (CPhCs) or opals, which renders significant flexibility in fabrication^11^,^12^,^13^,^14^.

PhCs have shown strong effect on fluorescence (FL) emission^6^,^15^,^16^. Depending on the relative position between the FL emission maxima and stopband minima, the PhCs can either inhibit^10^ or enhance emission^17^,^18^. Such a strong photonic modulation is not only exhibited on FL intensity, but also on emission lifetime, attributed to the localized density of states (LDOS)^7^,^19^,^20^.

PhCs offer two modes for FL enhancement^6^: 1) matching the leaky eigenmodes of PhCs with the fluorophore excitation wavelength (E) for enhanced excitation; 2) matching the leaky eigenmodes of PhCs with the emission wavelength (F) for enhanced light extraction. It has been consistently demonstrated both theoretically and experimentally that the enhanced excitation in Mode 1 results in higher gains in FL intensity compared to Mode 2^6^,^16^. A recent study^8^ showed a 320-fold luminescence enhancement of [Ru(dpp)_3_]Cl_2_ dispersed on a PMMA CPhCs that is in resonance with the excitation wavelength. Dual-stopband configurations with an excitation-in-resonance CPhCs (shorthand: E) stacked on top of an emission-in-resonance CPhCs (shorthand: F) has resulted in an almost twice as strong FL signal compared to monolithic E CPhCs^21^. The enormous fluorophore FL intensity enhancement induced by the CPhCs not only has strong application potential, but also raised interesting questions on its mechanisms.

Herewith, for the first time we report a double heterostructure CPhCs, stacked in a sequence of E-F-E, which has achieved all polymer unprecedented FL enhancement. Owing to this novel optical configuration, the Rhodamine B (RhB) FL enhancement was improved by almost a factor of 6× over monolithic CPhCs (E and F), reaching a thousand-fold enhancement compared to that of the same amount of RhB deposited on glass. The enhancement mechanism has been investigated by time-resolved FL spectroscopy.

Such a strong FL enhancement may find applications in sensing, light sources and optical data storage etc. Herein, we demonstrate the multi functionality of such a FL enhancement platform in sensing application. Being a macroporous material, the CPhCs self-assembled from polystyrene (PS) spheres can serve as an immobilization platform for the fluorescent sensing agents. Moreover, we can confine the fluorescent sensing agent in the middle F layer, in doing so the top and bottom E layers can function as protective membranes to the middle F layer. We illustrate this idea by replacing the RhB with carbon quantum dots (CDs) that can be used as Hg^2+^ ion sensors^22^.

## Materials and Methods

### Structure Design

**Figure 1:**
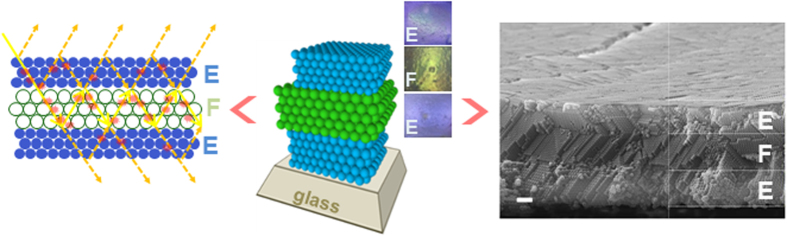
Left: Schematic of the coupling of photonic crystal effect and multiple-beam interference in the CPhCs E-F-E double heterostructure; Middle and Right: Illustration and SEM image of an E-F-E double heterostructure (Scale bar is 1 μm). Insets: top-view photo images of each layer.

The novel structure can be described as a double heterostructure 3D CPhCs structure in a sequence of E-F-E from bottom to top, as illustrated in Fig. 1, (where E refers to the monolithic CPhCs with a periodicity overlapping the excitation wavelength, and F refers to the monolithic CPhCs with a periodicity overlapping the emission wavelength). The thickness of the F layer is tuned to support constructive multiple beam interference of the excitation wavelength. The design rationale is based on the etalon configuration; in such a double heterostructure, the two E layers both reflect any excitation light that may propagate in the F layer. Hence, when the thickness of F supports constructive interference, the excitation light is effectively recycled many times through the F layer, increasing the excitation efficiency. Moreover, the cavity of this resonator, i.e. the F layer, itself is a CPhCs with a partial bandgap overlapping the fluorophore emission wavelength, which is a strong resonator of the emission light. This differentiates our E-F-E double heterostructure from the typical sandwich structures in which a layer of dye is cladded between two E or two F layers^23^ or sandwich structures without considering constructive interference^24^.

### Key Parameters

RhB dispersed in ethanol was selected as the emitter in our study. The FL emission of RhB was measured at different excitation wavelength, the RhB dispersion gives off the strongest FL emission at 610 nm (Figure S1) when the excitation wavelength is 460 nm. A Bragg diffraction model^25^ predicts that a partial bandgap occurs at:


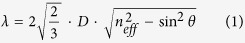


Where λ is the stopband wavelength, D is the diameter of the constituent spheres, and n_eff_ is the effective refractive index of the CPhCs structure and *θ* is the angle between the incident light and the lattice planes. The latter is estimated by 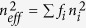, with constant filling fractions (*f*_*i*_) of 0.74 for PS phase and 0.26 for air. We therefore synthesized the E and F layers using PS colloids of 230 nm and 310 nm particle diameter to match the excitation and emission maxima of RhB.

### Synthesis and fabrication

Two different size of 230 ± 5 nm (E) and 310 ± 5 nm (F) polystyrene (PS) spheres were synthesized by an emulsifier free polymerization method^26^. A colloidal suspension was purified by several cycles of centrifugation. Monolithic E and F CPhCs were grown on micro glass substrates by horizontal self-assembly method. Double heterostructure CPhCs (E-F-E and F-E-F) were fabricated on the glass by sequential horizontal deposition method^27^. RhB was used as the model fluorescence dye, prepared at a concentration of 1 μM dissolved in ethanol. 20 μl of this solution was infiltrated into all different CPhCs structures. After drying in air, the fluorescence spectra of these dye-infiltrated CPhCs were measured.

To illustrate the difference in selecting the reference point for enhancement factor calculation, we have prepared a set of control samples for FL intensity comparison: 1) drop-casted film of 20 μL of 1 μM RhB in ethanol on a glass slide (short name: glass control), and 2) horizontal self-assembly of 230 nm PS spheres on the glass then heated at 120 °C for 2 h to melt and form an amorphous PS polymer (aPS) film. The same quantity, namely 20 μL of 1 μM of RhB was then drop-cast on the PS polymer film. This reference sample is referred to as the aPS control.

### Fabrication and testing of mercury ion sensing platform

PS spheres (PS) of 185 ± 5 nm (E) and 230 ± 5 nm (F) were synthesized for matching the excitation and emission wavelength of CDs^22^. CDs were immobilized on 230 nm PS spheres by a diffusion and entrapment method (CDs@F)^28^. In detail, PS suspension (5vol %) were first purified by several cycles of centrifugation. Then, 3.9 mmol CDs dispersed in dimethyl formamide (DMF) was added drop-wise to the 2 ml of washed 230 nm PS suspension. Finally, CDs immobilised PS spheres were washed and purified by several cycles of centrifugation to remove excess CDs. The double heterostructure were formed by successive self-assembly of E, F and E layers. Here, the E layers are CPhCs with PS spheres of 185 nm, and the F layer is a CPhCs with CDs entrapped, 230 nm size PS spheres.

### Characterization

PS particle hydrodynamic size and polydispersity were measured on Malvern Zetasizer Nano ZS, and particle size was determined by scanning electron microscope (SEM) using a JEOL 7001. The surface morphology of all CPhCs structures were examined by SEM, operating at 15 KV. UV visible (UV-Vis) absorption spectra were measured on Agilent 8453 UV-Vis spectrometer. Fluorescence emission spectra were recorded on Thermo Scientific Lumina fluorescence spectrometer at 600 V excitation power, to prevent bleaching of RhB. The incidence and observation light paths of the fluorescence spectrometer are perpendicular to each other, and the sample was placed in a position where both incidence and observation angles are of *θ *= 45° to the normal direction, as illustrated in Figure S2 in the Supporting Information. The laser spot on the sample was approximately 5 mm^2^ broad. The decay rate of the excited state of the RhB in the CPhCs was measured on an Edinburgh Photonics FLS920 with 450 nm 100 ps pulsed laser excitation source.

## Results and Discussions

The double heterostructures were fabricated by horizontal deposition^29^ combined with doctor blading method on glass slides, covering an area of 2 cm × 2 cm. The cross-sectional SEM image of a typical E-F-E structure, displayed in Fig. 1, clearly shows the double heterostructure configuration and the congruent interfaces. SEM image in Figure S3 in Supporting Information reveals that the top E layer exhibits a high density of cracks with an average domain size of c.a. 20–30 μm; on the contrary the E monolayer on glass substrate prepared by the same method of the same thickness has no cracks over a size of close to mm, although there are visible crystal misalignments and grain boundaries, as shown in Figure S3 (b). The difference in the presence of cracks between the monolayer and multilayer structures is likely caused by the better mobility of PS spheres on the treated glass surface during drying^30^,^31^ in contrast to the inhibited mobility on the uneven underlying substrate, i.e. the F-E structure. The fact that the E layer particle size (230 ± 5 nm) is smaller than the underlying F layer particle size (310 ± 5 nm) further decreases the mobility of E particles, because the smaller particles can be locked in the tetrahedral sites formed by the larger particles in the F layer.

**Figure 2:**
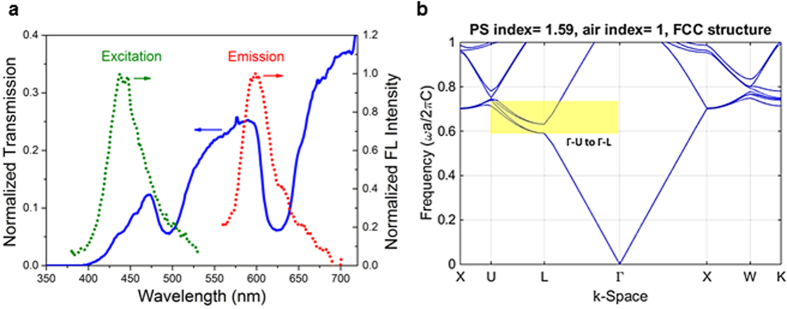
(**a**) UV-vis transmission of double heterostructure E-F-E at an incident angle of 25° overlaid with the normalized fluorescence excitation and emission spectra of RhB in E-F-E. (**b**) Band diagram calculated by plane-wave expansion.

Figure 2a shows the dual stopbands of E-F-E structure (thickness of each layer is 2.5 μm) and their relative positions with reference to the excitation and the emission of RhB. The spectra pattern of the dual stopbands suggests that the morphology defects in the top E layer do not appear to have a noticeable influence on the optical performance of E-F-E CPhC superstructure. It is shown that the RhB excitation peak is on the blue edge of the E stopband, and that the emission peak falls on the blue edge of the F stopband. It’s understood that when the absorption of a material overlaps the stopband blue edge, there would exist an enhanced light-matter interactions due to the LDOS in the solid phase of PhCs^32^, and that when fluorophore emission peak is on the blue edge of the PhC bandgap there would be an enhanced emission intensity accompanied by an accelerated emission rate^7^.

The calculated bandgap of a standard Face-Centre-Cubic photonic crystal made by PS sphere close packing shown in Fig. 2b is consistent with the experimental observation. The calculation is based on plane-wave expansion with a refractive index of 1.59. Since in fluorescence measurement, the incidence and observation light paths are perpendicular to each other (Figure S2), at the incident of 25 degree, the observation light path is not at the standard reflection position, which corresponds to between the range of Γ-L and Γ-U direction in the reverse space. For PS spheres of 230 nm, the bandgap is centred at 470 nm; and for PS spheres of 310 nm, the bandgap is centred at 630 nm when the corresponding normalized frequency is 0.69.

**Figure 3:**
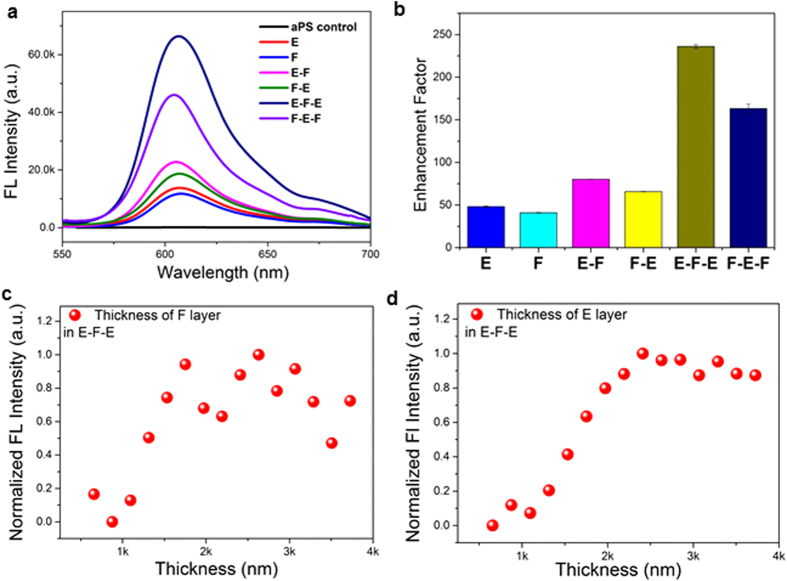
(**a**) FL spectra for RhB-infiltrated monolithic 3D CPhCs of E and F, heterostructures of E-F and F-E and double heterostructures of E-F-E and F-E-F. Control is RhB on aPS. (**b**) FL enhancement factors for all samples, measured as the ratio of FL intensity maxima between the sample and the control. (**c**) The effect of F layer thickness on RhB FL intensity in E-F-E double heterostructure; (**d**) The effect of E layer thickness on RhB FL intensity in E-F-E double heterostructure.

Figure 3(a) shows the FL spectra of RhB in E-F-E, in monolithic E and F, and heterostructures E–F and F-E (all infiltrated with the same quantity of RhB dye) and the enhancement factors measured relative to the aPS control sample (Fig. 3(b)). The evaluation of FL emission enhancement in CPhCs requires a control sample that ideally should have the same structural and chemical environment as the specimen of interest. In this study, we selected RhB spread on the aPS film of the same thickness as the reference, because it enables us to cancel out the effect of RhB-PS molecular interactions. Nevertheless, to benchmark against other reported results, e.g. in^8^, the FL enhancement factors relative to RhB on glass are presented in Figure S4, which shows a 1031-fold increase for E-F-E.

In the monolithic E CPhCs, the excitation light is coupled to the stopband, leading to enhanced excitation, while in the monolithic F CPhCs, it is the emission light that is coupled to the stopband. The quality factors of the two types of monolithic CPhCs are similar, 40-fold enhancement (compared to aPS control) with E slightly better than F, consistent with other reports^6^,^21^.

The E-F-E double heterostructure exhibited a remarkably high FL enhancement factor of 236 relative to RhB on aPS, which is more than five-fold of that of the monolithic E and F CPhCs. Such an intensity enhancement effect would appear almost surprising, because with an E layer on top and the incident light shone from the top, intuitively one would expect that very little excitation light (about 8% according to the UV-vis transmission data in Fig. 2a in this case) would pass through E layer and enter into F layer. However, as we will show, it is a doubly resonant effect that causes such strong intensity enhancement in our case. The additional enhancement in the E-F-E double heterostructure arises because the two E layers form an optical resonator for the excitation light, enhancing the excitation intensity in the F layer and thus the fluorescence intensity. As expected by the multiple beam interference theory, the RhB FL intensity in E-F-E is the highest when the F layer thickness supports constructive interference of excitation wavelength. The undulating pattern of the RhB FL intensity when F layer thickness varies in E-F-E structure as shown in Fig. 3(c) confirms the multiple-beam interference effect. In contrast, when F layer thickness varies in a monolithic CPhCs, the FL intensity only increases in a monotonic manner as shown in Figure S5. Moreover, as shown in Fig. 3(d), when the thickness of E layer increased, it initially resulted in improved enhancement in FL intensity of E-F-E until reaching a maximum before showing a gradual decreasing trend. This can be understood as when the thickness of E layer increases, initially its strength as a Bragg’s reflector increases though the transmission of the excitation light decreases, and the gain in FL intensity benefits more from the improved multiple beam interference compared to the loss in transmitted light through top E layer; such a gain in Bragg’s reflector strength accompanied by a loss in transmission through E means that there exists an optimum thickness for emission intensity as demonstrated in Fig. 3(d).

Basic resonator theory predicts an enhancement factor of *S*_*EFE*_ = *S*_*F*_/*T*_*E*_, where *T*_*E*_ is the stopband transmission of the E layer, approximately 7.9% from Figure S6(a). However, scattering losses in the F layer reduce the enhancement factor^33^ to:


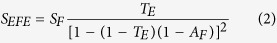


Where 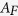 is the fraction of intensity lost in a single pass through the F layer. Figure 2a shows that the E-F-E double heterostructure, which is two times thicker than the F layer alone, has a passband transmission that is 3% lower than the F layer. Hence 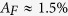, theoretically yielding about 9× enhancement compared to F layer alone. The reason that only 6× enhancements (relatively to F layer) was experimentally observed is likely due to scattering loss and the imperfection of the CPhC superstructure. The F-E-F double heterostructure exhibits a lower enhancement of 4× relative to E layer alone, arising from similar multiple-beam interference effects, this time for the fluorescence light. In Fig. 2, the scattering loss at the fluorescence wavelength appears slightly larger than at the excitation wavelength, which may account for this effect.

**Figure 4:**
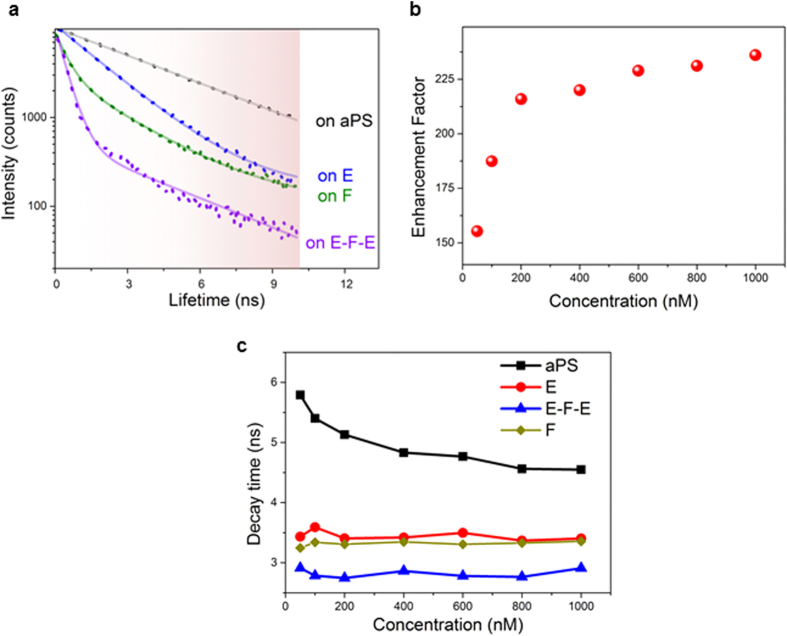
(**a**) FL decay curves for RhB infiltrated CPhCs and aPS. (**b**) The enhancement factor of RhB on double heterostructure E-F-E at 25° with different RhB concentrations. (**c**) Averaged lifetime constants of RhB on aPS, on monolithics CPhCs E and F, and on double heterostructure E-F-E at 25° with different RhB concentrations.

We further investigated the effect of E-F-E double heterostructure on the FL emission lifetime to understand the origin of such a strong FL enhancement. It has been demonstrated that PhCs can modify the radiative lifetime due to the redistribution of DOS^10^, which is even observable in PhCs of low refractive index contrast^19^,^34^ but more noticeable when the refractive index contrast is sufficiently high and a full bandgap arises^7^,^20^. Since the RhB PL intensity in E-F-E is the highest at the incident angle of 25°, we selected this orientation for detailed FL dynamics analysis. The RhB emission decay curves of RhB in aPS control, monolithic E and F CPhCs, and double heterostructure E-F-E at the incident angle of 25° are shown in Fig. 4(a). Apart from RhB on aPS, the decay curves of RhB in all CPhC structures show typical nonexponential time-resolved emission observed in PhCs^35^, which can all be fitted with a biexponentail function^36^. The lifetime constants and percentages are displayed in Table S1 in Supporting Information. Emission of RhB in monolithic E CPhC appears to have two emissive components: one with a lifetime constant of 3.83 ns and a percentage of 13.3%, likely arising from the same origin as RhB on aPS (lifetime 4.59 ns), and one faster emission component with a lifetime constant of 1.77 ns contributing 86.70% of the total photon counts. RhB in F shows a similar dynamics with a slow emission (3.7 ns, 29.9%) sharing the same origin as RhB on aPS, and a fast emission (0.58 ns, 70.1%) likely due to photonic structure. The difference between RhB in E and F is that the emission in F is much further accelerated than RhB in E. Such an outcome is plausible, because at the incident angle of 25°, the intrinsic FL of RhB overlaps the blue edge of the stopband of F; it has been shown that such a coupling results in much enhanced radiative rate^18^,^37^,^38^. On the other hand, the shorter lifetime component observed in E is still under investigation. Studying fluorophore emission dynamics in E type of CPhCs with bandgap overlapping the excitation wavelength of fluorophores is relatively a novice; strong deviation in measured lifetimes from the reference sample has been reported by Song and co-workers^8^, however they observed a slowed dynamic in comparison to the fluorophore on glass. Fitting of the emission decay curve of RhB in E-F-E shows two emission components with a long lifetime of 4.1 ns (5%), similar to RhB on aPS and a short one of 0.41 ns (95%). It is an extraordinary four-fold acceleration compared to τ_2_ of RhB in E.

The two components of emission observed from time-resolved FL analysis fit well with the static FL spectra. A small peak cantered around 630 nm can be distinguished on the angle-dependent RhB static emission spectra by performing peak-splitting (Figure S8). The spectral position of the small peak coincides with the emission maxima of RhB on aPS, which suggests that this component of emission originates from the RhB dye on the very top of the double heterostructure. The intensity of double heterostructure CPhCs is moderately enhanced due to coupling with the leaky mode, however, without changes in emission lifetime, similar to the system reported by Ganesh *et al.*^6^. On the contrary, the major peak centred around 590 nm appears to originate from the RhB dyes inside CPhCs, which is more sensitive to the change of incident angle. It is the lifetime of this emission component that is significantly changed by the photonic effect, consistent with the quantity contribution in collected photons in lifetime measurements.

To further verify whether the FL modulation is due to photonic effect, we also studied the dependence of the enhancement factor and decay time constant on the concentration of RhB molecules. The original RhB solution was diluted to different concentrations with ethanol. As shown in Fig. 4(b), the enhancement factor increased with RhB concentration increase from 160 times to 236 times. When the concentration increased from 0 to 200 nM, there was a rapid FL increment. When RhB concentration increased further to 1 μM, the increase rate of FL was considerably slower. In the decay time comparison as shown in Fig. 4(c), the averaged decay times for RhB in various CPhCs were used. The decay constant of RhB on aPS rapidly decreased from 5.8 ns to 4.8 ns when RhB concentration increased from 10 nM to 400 nM, followed by a stabilized regime with an average of approximately 4.6 ns. Such a decrease in FL lifetime is likely caused by the energy transfer among dye molecules as the concentration increased. On the contrary, for RhB in E, F, and E-F-E, the emission lifetime remained constant at all test dye concentrations, further confirming that the observed modified lifetimes in CPhC structures are due to the photonic effect, not dye aggregations. The fluorescence enhancement enables us to detect the presence of RhB with ultra-sensitivity. The detection limit of RhB in E-F-E decreased from 37 pM on glass to 18 fM, more than three orders of magnitude reduction (Figure S9).

**Figure 5:**
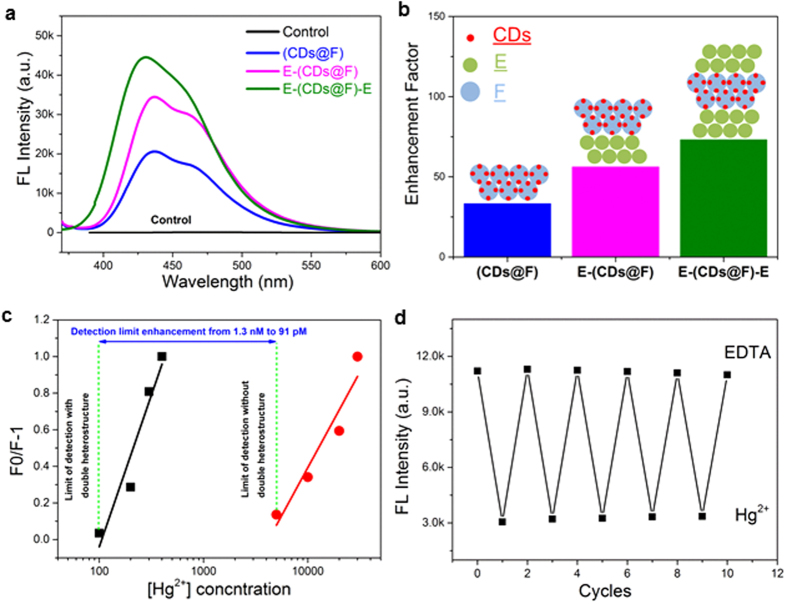
(**a**) The fluorescence spectra double heterostructure E-(CDs@F)-E, heterostructure of E-CDs@F, and single monolithic (CDs@F), control is CDs infiltrated into the amorphous PS structure. (**b**) FL enhancement factor for all samples, measured as the fluorescence intensity ratio between the sample and the control. (**c**) concentration-dependent FL linear response of E-(CDs@F)-E film sensor by of Hg(II) compared to concentration-dependent FL linear response of CDs. (**d**) Reversible changes of the FL intensities of the film sensor when immersed in the solution of 1.0 nM Hg^2+^ and 2 mM EDTA alternately.

We further demonstrate its function as a fluorescence sensing enhancement platform by coating mercuric ion-sensitive CDs on the PS beads of F layer and forming E-(CDs@F)-E double heterostructure. The simple swelling-diffusion method resulted in stable immobilization of CDs on the outer layer of PS spheres with preserved sensitivity to Hg (II). Such an E-F-E configuration shows enhanced signal compared to monolithic CPhCs and heterostructure as shown in Fig. 5(a). As shown in Fig. 5(b), 73-fold FL enhancement was observed for E-(CDs@F)-E, whereas a 56-fold enhancement for E-(CDs@F) heterostructure and 33-fold enhancement for monolithic (CDs@F) was observed. Interestingly, a different enhancement factor was obtained for RhB and CDs which might be attributed to the changed vibration bonds, the varied energy transfer rate and the variant polarity of the CDs and RhB molecules^8^. Figure 5(c) shows an ultrasensitive sensitivity to Hg(II) with a detection of limit of 91 pM, 14-fold lower than the detection limit of CDs as suspension (1.3 nM)^22^, owing to the employment of E-F-E platform.

Moreover, such a cladded structure provides excellent protection to the sensing agents, namely the CDs coated on the surface of F PS spheres; the two E layers at the top and bottom act as macroporous filters for analyte samples as well as irradiation protection. The sensing device shows excellent reversibility to mercuric ion binding, as illustrated in Fig. 5(d). It can be envisioned that if the double heterostructure are made into μm size, incorporating surface patterning to promote analyte concentration^39^, the sensitivity and accuracy can be even further heightened.

## Conclusions

We have shown that an E-F-E double heterostructure CPhC can enhance the directional emission of optically excited fluorescence to an unprecedented extent for a purely polystyrene-based thin film. The outer E-layers of the double heterostructure act as an optical resonator for the excitation light, enhancing the excitation rate of fluorophores in the central F-layer. At the same time, the F-layer leaky mode is in resonance with the fluorescence emission. The coupling of the two resonance modes is the cause of the significant fluorescence intensity enhancement, in relation to E and F monolithics.

The emission dynamics study confirms that the significant FL enhancement is due to photonic effect, and that the E-F-E double heterostructure induces extraordinary emission acceleration, resulting in a lifetime constant of 0.41 ns in comparison to 1.77 ns found in monolithic E structure. Such a 4.3-fold emission enhancement is remarkable.

Our E-F-E type of double heterostructure CPhCs, which can be fabricated through facile self-assembly route with inexpensive materials, should find immediate applications in illumination, light harvesting, laser and chemical/biochemical sensing. The Hg (II) sensing application shown in this study further demonstrated that such a novel architecture for FL enhancement provides multifunction for solid-state optical sensors.

## Additional Information

**How to cite this article**: Eftekhari, E. *et al.* Anomalous Fluorescence Enhancement from Double Heterostructure 3D Colloidal Photonic Crystals–A Multifunctional Fluorescence-Based Sensor Platform. *Sci. Rep.* **5**, 14439; doi: 10.1038/srep14439 (2015).

## Supplementary Material

Supplementary Information
