# Supplementary material for: Anomalous Fluorescence Enhancement from Double Heterostructure 3D Colloidal Photonic Crystals–A Multifunctional Fluorescence-Based Sensor Platform
Source: Sci Rep. 2015 Sep 24;5:14439. doi: 10.1038/srep14439 (PMC4585865; doi:10.1038/srep14439)
Supplement: Supplementary Information [file srep14439-s1.doc]

Anomalous Fluorescence Enhancement from Double Heterostructure 3D Colloidal Photonic Crystals - A Multifunctional Fluorescence-Based Sensor Platform

Ehsan Eftekhari^1^, Xiang Li^1^, Tak H. Kim^1^, Zongsong Gan^5^, Ivan S. Cole^2^, Dongyuan Zhao^3^, Dave Kielpinski^4^, Min Gu^5^, Qin Li^1*^

1. Queensland Micro- and Nanotechnology Centre & School of Engineering, Griffith University, Nathan, QLD 4111, Australia

2. CSIRO Materials Science and Engineering, Clayton, VIC 3168, Australia

3. Department of Chemistry & Laboratory of Advanced Materials, Fudan University, Shanghai, 200433, P.R. China

4. Centre for Quantum Dynamics, Griffith University, Nathan, QLD 4111, Australia

5. Centre for Micro-Photonics and CUDOS, Swinburne University of Technology, Melbourne, Hawthorn VIC 3122, Australia

*Corresponding Author: qin.li@griffith.edu.au

Address: Queensland Micro- and Nanotechnology Centre & School of Engineering, Griffith University, Nathan, QLD 4111, Australia

Telephone: (61)(07)37357514

**S1. Rhodamine B (RhB) absorption and reference spectra**

**S2. Fluorescence spectrometer setup**

**S3. Morphology of top E layer in E-F-E and E layer on a glass substrate**

**S4. FL enhancement factors when compared with different control samples**

**S5. Effect of F thickness on FL in monolithic CPhCs**

**S6. Transmission overlaid with FL excitation and emission spectrum of RhB**

**S7. Fluorescence lifetime**

**S8. Peak splitting for FL spectra**

**S9. Detection limit**

**S1. Rhodamine B (RhB) absorption and reference spectra**

Figure S1 displays the fluorescence spectra of Rhodamine B (RhB) obtained from Thermo Scientific Lumina fluorescence spectrometer at 600 V excitation power. The left panel shows the fluorescence spectra of RhB solution, while the right panel shows the fluorescence spectra of RhB in solid state as a thin film on glass substrate.


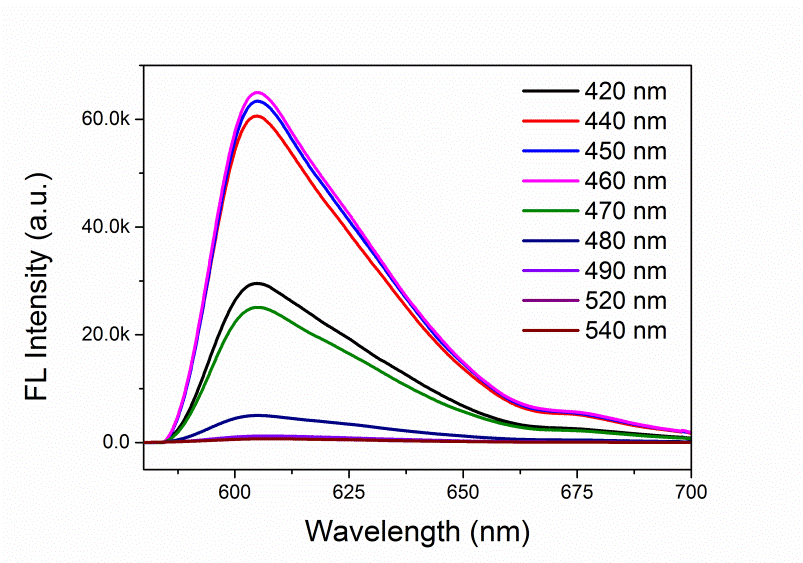

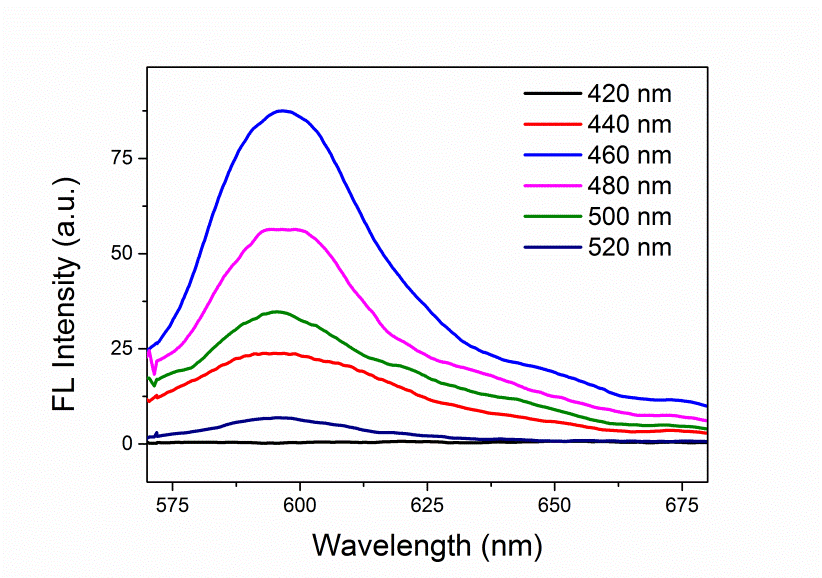


**a b**

Figure S1 (a) The FL spectra of RhB in ethanol with a concentration of 1 µM. (b) The FL spectra of RhB deposited on glass from the same solution as a control sample.

**S2. Fluorescence spectrometer setup**


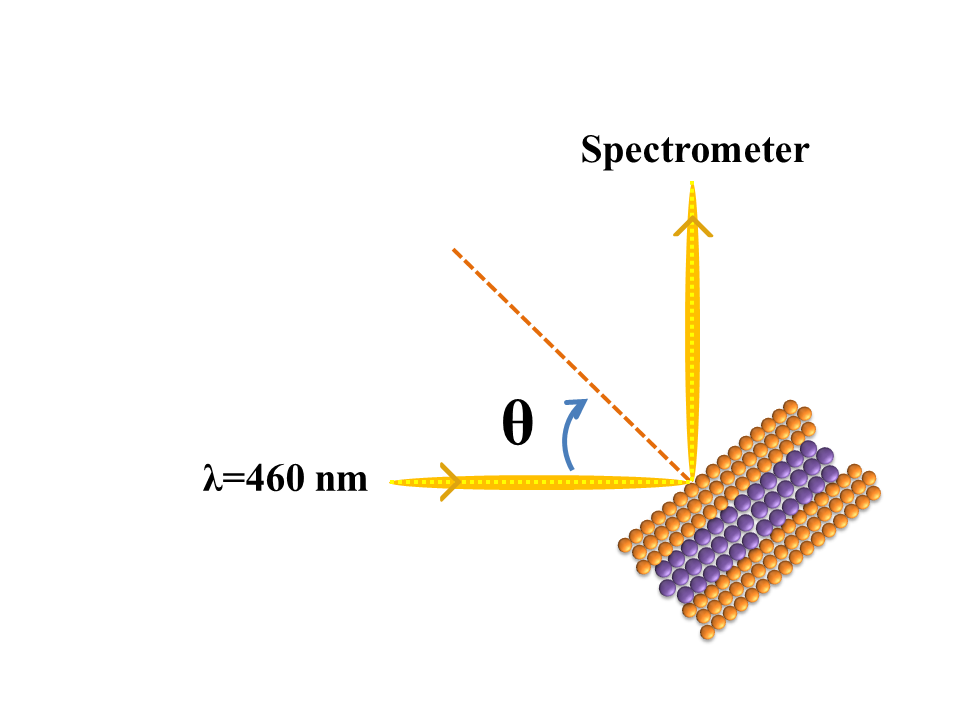


**Figure S2** Illustration of the fluorescence spectrometer configuration.

**S3. Morphology of top E layer in E-F-E and E layer on a glass substrate**


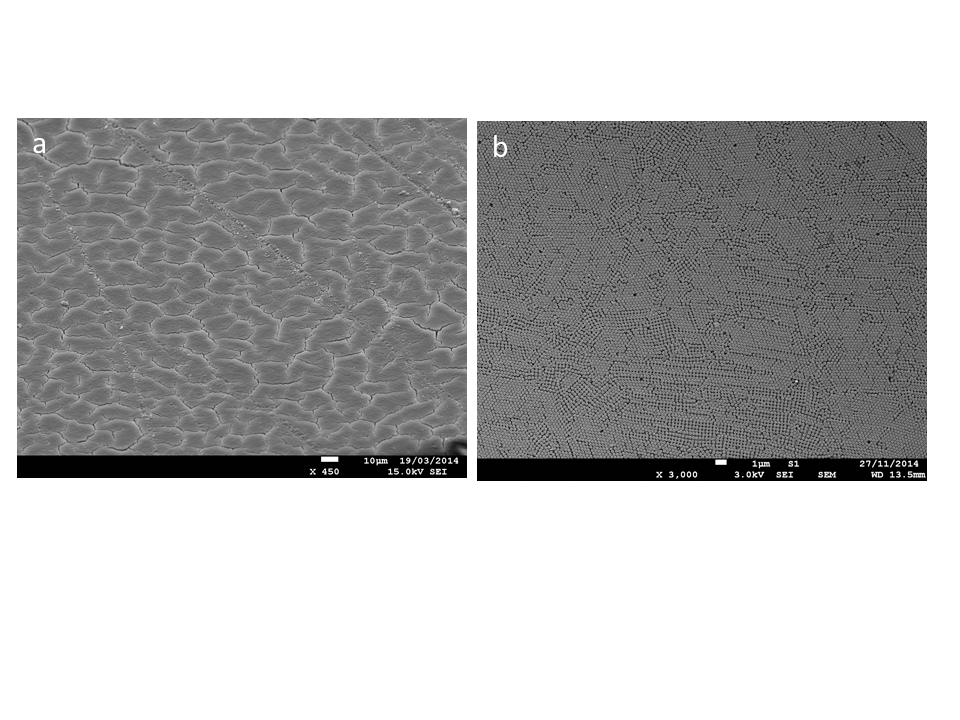


**Figure S3** (**a**) SEM images of the top E layer in E-F-E double hetereostructure (scale bar: 10 µm) (**b**) and E layer on glass substrate (scale bar: 1 µm).

**S4. FL enhancement factors when compared with different control samples**


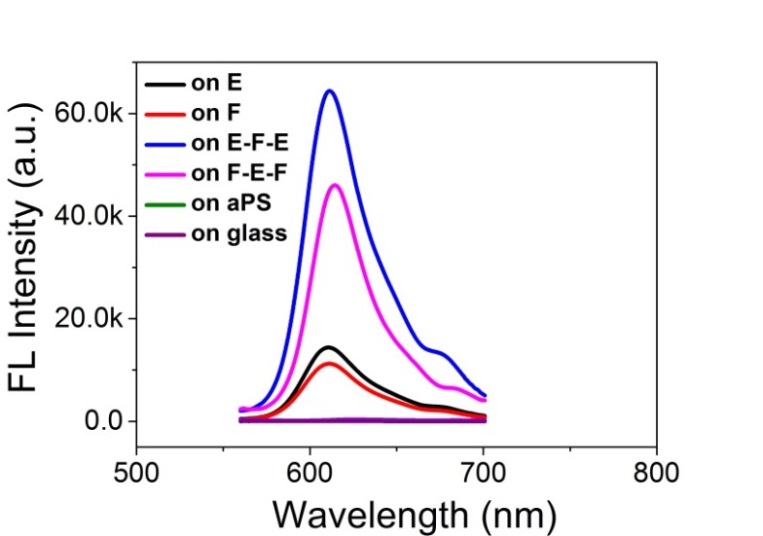

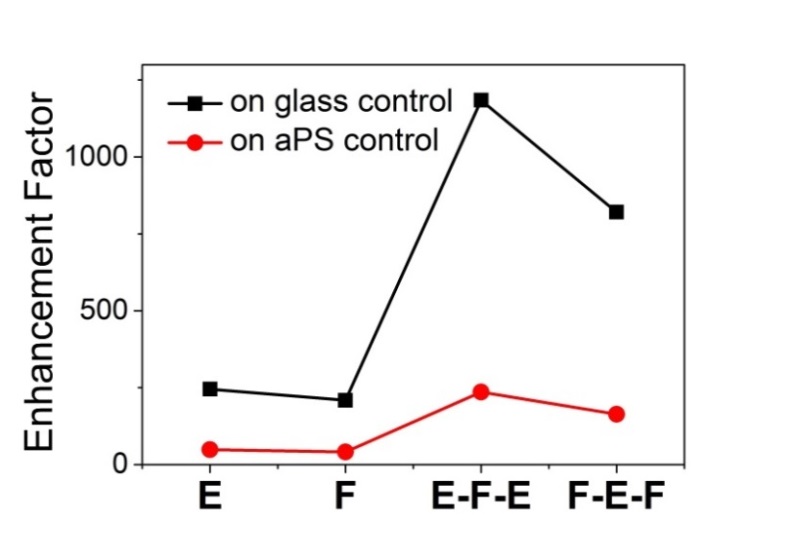


**a**

**b**


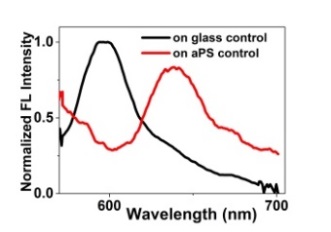


**Figure S4** (**a**) FL spectra for E,F and double heterostructure E-F-E and F-E-F compare to two control samples of RhB on amorphous PS (aPS) and RhB on the glass (glass control). Inset is the normalized FL spectra of glass and aPS control samples. (**b**) The fluorescence enhancement factors when E, F and double heterostructre E-F-E and F-E-F compared with glass and aPS control samples.

**S5. The effect of F thickness on FL in monolithic F CPhCs**


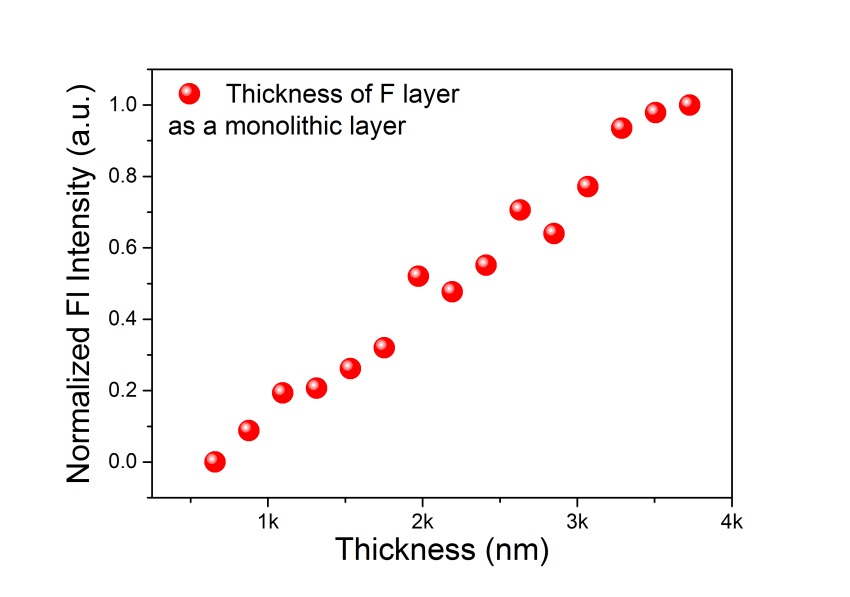


**Figure S5** shows the monotonic FL increase when the thickness of F monolithic CPhCs increases.

**S6. Transmission overlaid with FL excitation and emission spectrum of RhB**


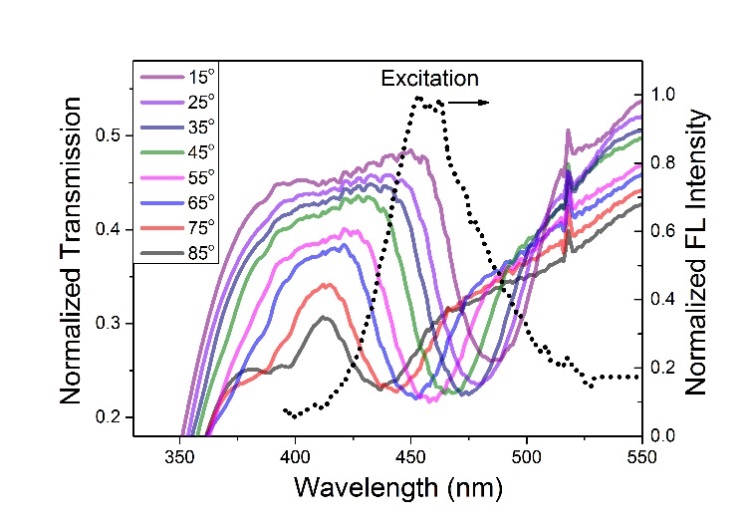

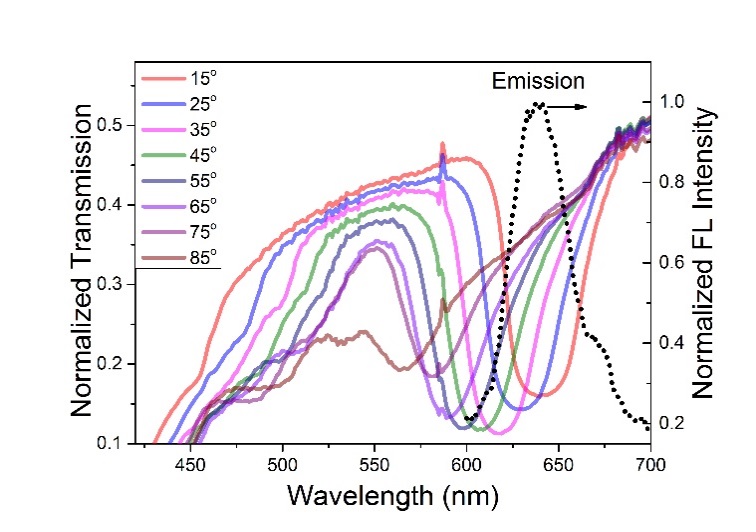


**a**

**b**

**Figure S6** (**a**) Normalized UV-vis transmission of monolithic E CPhCs as a function of incident angle overlaid with normalized fluorescence excitation spectrum of RhB. (**b**) Normalized UV-vis transmission of monolithic F CPhCs as a function of incident angle overlaid with normalized fluorescence spectra of RhB emission.

**S7. Fluorescence lifetime**

The fluorescence decays of RhB inside the CPhCs at 25˚ incident angle were measured using a time-resolved single photon count fluorescence spectrometry (Edinburgh Photonics FLS920 fluorescence spectrometer with 450 nm pulse laser excitation source). The decay curve of RhB on aPS was fitted with a biexponential function, following$I(t)=\sum_{i} a_{i}e^{\frac{-t}{\tau_{i}}}$. The percentages of each fraction are calculated by$a_{i}/\sum_{i} a_{i}$. The results are shown in Table S1.

Table S1. Time-resolved fluorescence parameters of RhB in the presence of CPhCs

|  | **Τ1 (ns)** | | **Rel (%)** | **T2 (ns)** | | **Rel (%)** | **R^2^** |
| --- | --- | --- | --- | --- | --- | --- | --- |
| **RhB on aPS** | 4.59 | 100 | |  |  | | 0.99 |
| **RhB on E** | 3.83 | 13.30 | | 1.77 | 86.70 | | 0.99 |
| **RhB on F** | 3.7 | 29.9 | | 0.58 | 70.1 | | 0.99 |
| **RhB on E-F-E** | 4.1 | 5 | | 0.41 | 95 | | 0.98 |

**S8. Peak splitting for the static emission spectra**


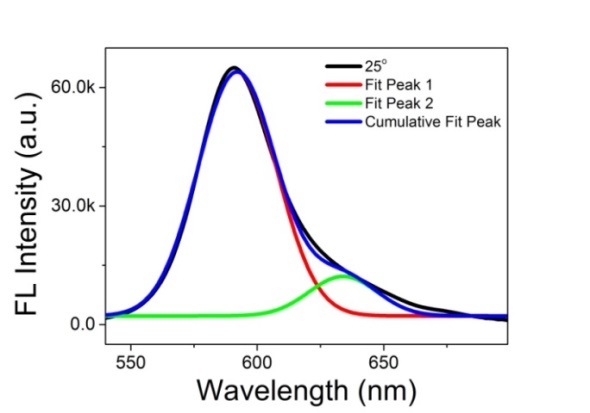

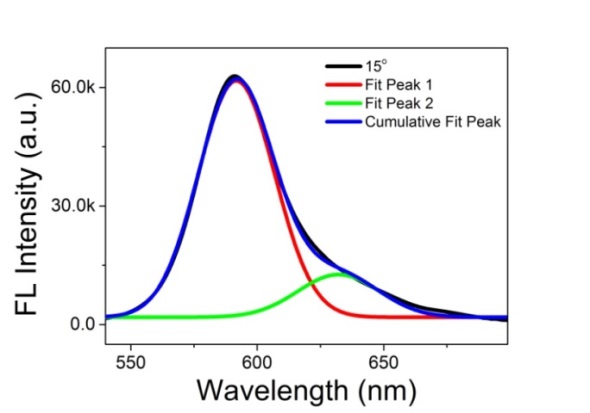

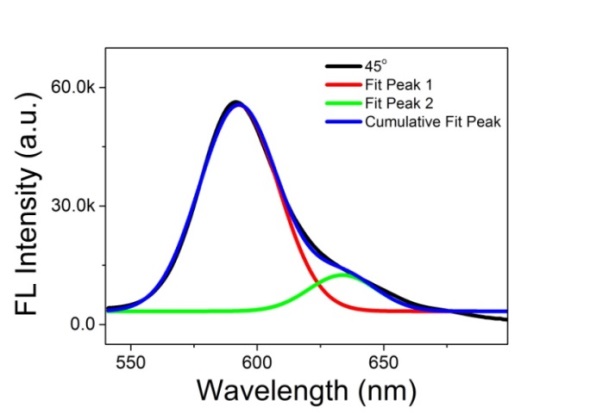

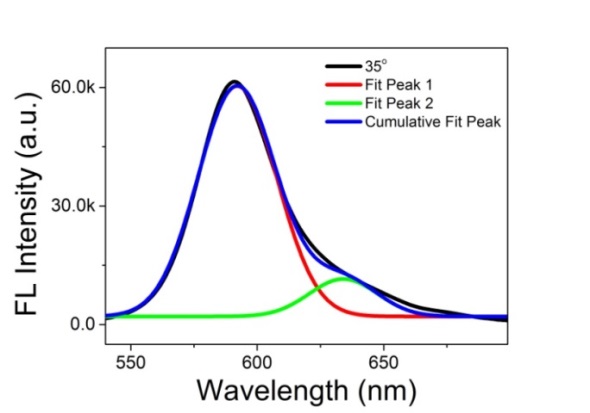

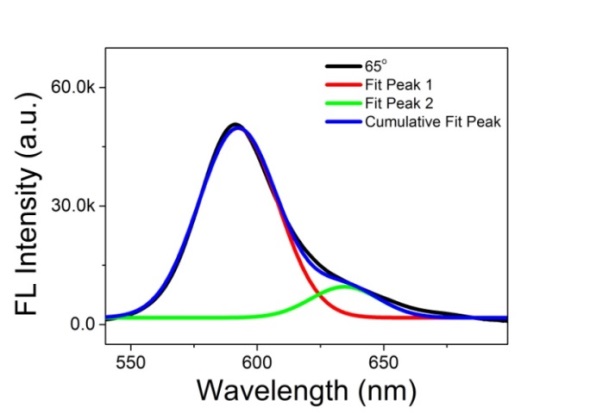

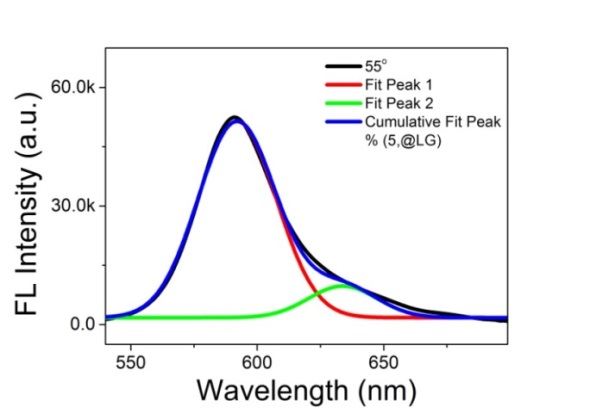

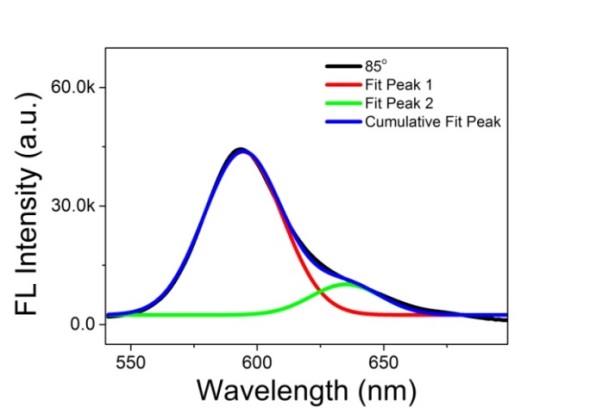

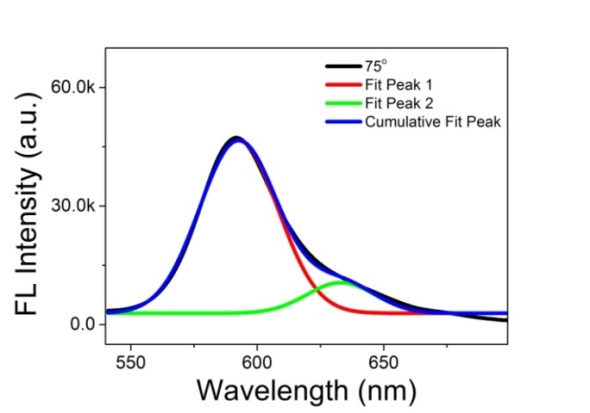


**Figure S8** Shows that the static FL spectra at all angles can be split into two peaks, indicating at least two types of emissions.

**S9. Detection limit of RhB**


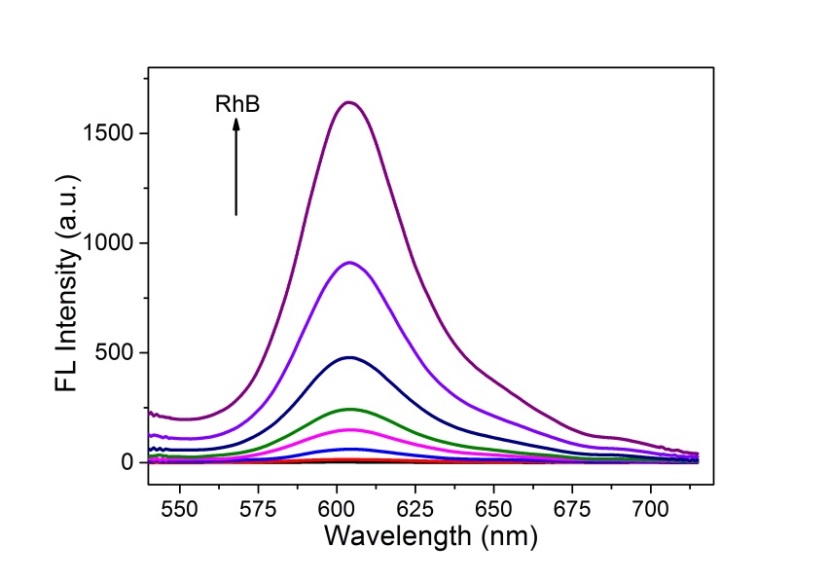

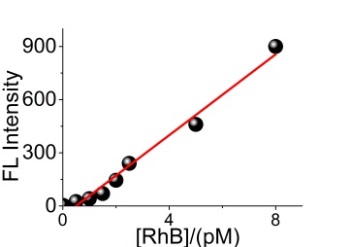


**b**


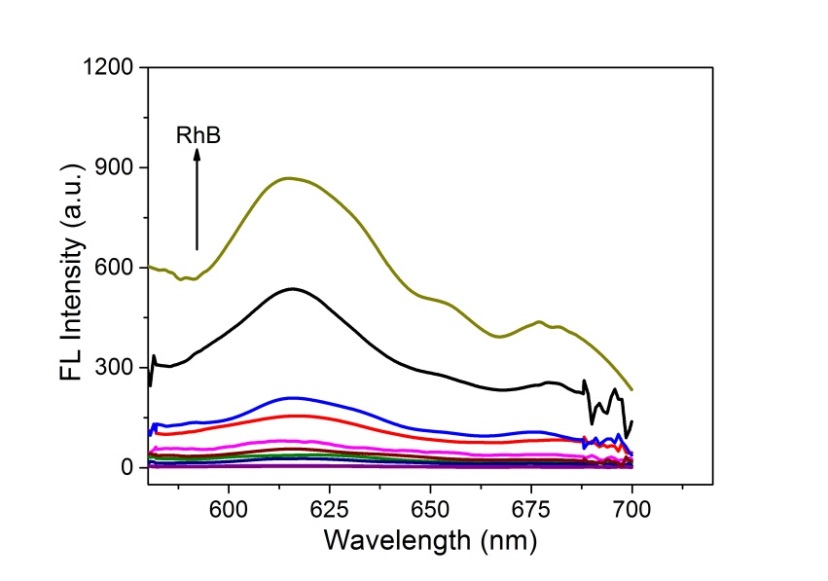

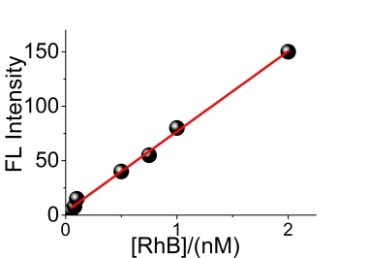


**a**

**Figure S9** (**a**) The FL signal of RhB of various concentrations deposited on glass stubstrates. Inset: concentration-dependent FL linear regression; (**b**) The FL signal of RhB of various conentrations deposited on the E-F-E film. Inset: concentration-dependent FL linear regression.

The linear relationship between the fluorescence intensity and the concentration of RhB was obtained by data regression analysis:

*Control: F_c_* **=** 73.64 * *C*_RhB_ + 3.15 (R^2^= 0.9607)

*Double heterostructure E-F-E*: *F_s_* **=** 114056 * *C*_RhB_ – 57.05 (R^2^= 0.982)

Where *F_c_ and F_s_* are the FL intensity of RhB for control sample and doubel heterostructure film, respectively; *C_RhB_* is the concentration of RhB; and R^2^ is the correlation coefficient. The detection limit, DL, of RhB was calculated with the following equation:

DL = 3σ/m

where σ is the standard deviation, and m is the slope of fit curve of the fluorescence intensity FL *vs* RhB concentration. For the control sample: the m value is 73.64, and σ is 0.9 nM. The DL is thus calculated to be 37 pM. For the E-F-E film, the m value is 114056 and σ is 0.69 nM. The DL is thus calculated to be 18 fM.
